# Data Mining Evidences Variabilities in Glucose and Lipid Metabolism among Fish Strains: A Case Study on Three Genotypes of Gibel Carp Fed by Different Carbohydrate Sources

**DOI:** 10.1155/2023/7589827

**Published:** 2023-02-02

**Authors:** Xuerong Song, Haokun Liu, Junyan Jin, Dong Han, Xiaoming Zhu, Yunxia Yang, Shouqi Xie

**Affiliations:** ^1^State Key Laboratory of Freshwater Ecology and Biotechnology, Institute of Hydrobiology, Chinese Academy of Sciences, Wuhan 430072, China; ^2^University of Chinese Academy of Sciences, Beijing 100049, China; ^3^The Innovative Academy of Seed Design, Chinese Academy of Sciences, Beijing 100101, China

## Abstract

An 8-week feeding trial was conducted to evaluate the application of common carbohydrate sources, cornstarch (CS), wheat starch (WS), and wheat flour (WF), to different gibel carp genotypes, Dongting, CASIII, and CASV. The results of the growth and physical responses were analysed by data visualization and unsupervised machine learning. As revealed by a self-organizing map (SOM) and the cluster of growth and biochemical indicators, CASV had superior growth and feed utilization and better regulation of postprandial glucose, followed by CASIII, while Dongting showed a high level of plasma glucose with poor growth performance. CS, WS, and WF were differently utilized by the gibel carp, and WF was associated with greater zootechnical performance based on higher specific growth rate (SGR), feed efficiency (FE), protein retention efficiency (PRE), and lipid retention efficiency (LRE), induced hepatic lipogenesis, increased liver lipids, and enhanced muscle glycogen. Spearman's correlation analysis of the physiological responses indicated that plasma glucose had a significantly negative correlation with growth, feed utilization, glycogen storage, and plasma cholesterol level, and it was positively related to liver fat content in gibel carp. Transcriptional variabilities were observed: CASIII showed increased expression of *pklr*, which is involved in hepatic glycolysis, and *pck* and *g6p*, which are involved in gluconeogenesis. Interestingly, Dongting showed upregulation of genes involved in glycolysis and fatty acid oxidation in muscle. Furthermore, there were numerous interactions between carbohydrate sources and strains for growth, metabolites, and transcriptional control, confirming the existence of genetic polymorphisms in carbohydrate use in gibel carp. Globally, CASV showed relatively better growth and carbohydrate utilization, and wheat flour seemed to be more efficiently utilized by gibel carp.

## 1. Introduction

Carbohydrates are regarded as the least expensive energy source in artificial feed. However, the optimal levels of dietary carbohydrates for fish are usually lower than those of other domestic animals due to their poor ability to regulate glucose homeostasis after carbohydrate intake [1]. In the past two decades, the rise in aquaculture has forced the reevaluation of carbohydrate utilization even though it is considered dispensable for fish growth [2]. Different types of carbohydrates were not equally utilized, and they varied greatly between species. Carnivorous fish such as white sturgeon (*Acipenser transmontanus*) could better use maltose or glucose than dextrin, raw cornstarch, or sucrose diets [3]. Gibel carp (*Carassius gibelio*), as a representative omnivorous fish, made better use of starch than glucose [4, 5], and the herbivorous fish grass carp (*Ctenopharyngodon idella*) also used more polysaccharides than monosaccharides [5]. Surprisingly, the sources of starch even differed across different varieties and had different effects on quality. Although a few studies have compared the effects of starch types on fish growth [6–9], the mechanism underlying the transport and metabolic pathways of glucose after starch source uptake is still unknown. With the development of molecular biology methods, it is possible to investigate how carbohydrate sources are taken in, transported, and metabolized differently in the fish body and to deeply understand the differences in the utilization of carbohydrate sources from the molecular level to the phenotype level.

The liver is the principal organ in the regulation of intermediary metabolism [10, 11]. Regarding glycometabolism, glucose is taken from blood by specific glucose transporters (*slc* family, previously named *gluts*) and then catabolized by glycolysis to provide ATP; glucose in excess could be stored as glycogen or converted into other metabolites, and gluconeogenesis also contributes to glucose homeostasis under food deprivation [12]. However, gluconeogenesis is still active in fish liver even when fed to satiation or fed high carbohydrate diets, which is regarded as one of the key reasons for postprandial hyperglycaemia in fish [1, 13]. Lipid metabolism is closely related to glucose metabolism. Hepatic lipogenesis was proven to be an efficient pathway for consuming glucose in response to glucose intolerance [1, 14, 15]. In addition, tissues other than the liver could also be essential for glucose utilization, such as the muscles, the largest part of the fish body, which has a higher energy demand. Moreover, the intestine plays a decisive role in carbohydrate digestion and glucose transportation at the initial step. While few studies have focused on nutrient metabolism in these two tissues, the changes in glucose-associated metabolism affected by dietary carbohydrates in fish muscle and intestine have not yet been fully discussed.

In recent years, many studies have been conducted on fish genotype selection in combination with nutritional indicators for selecting genotypes with better growth and feed utilization. In rainbow trout, diverse characteristics in response to diets and conditions between isogenic lines have been observed [16–18]. CASIII and CASV are strains (varieties) obtained by artificial selection using the gynogenesis technique [19]. Unlike the wild type (Dongting), which feeds on natural bait, fish in the artificial selection process are fed artificial feed, so the characteristics of using artificial feed are continuously enhanced during the selection process. Previous studies have shown that CASV has a higher protein retention rate [20, 21]. In addition, different changes in glucose and lipid metabolism between gibel carp strains were also found after insulin or glucose treatment [22, 23]. Carbohydrates are regarded as cost-effective energy in artificial aquafeeds, but the correlation of dietary carbohydrates and physical responses, as well as the associations of these results from different aspects, is not well understood. The present study objectively revealed the specificity between genotypes and their preference for specific diets through machine learning techniques. For example, a self-organizing map (SOM) and Spearman's correlation coefficient (*r*) were used to explore the relationship between growth, feed efficiency, and postprandial metabolites across gibel carp genotypes and carbohydrate sources. Principal component analysis (PCA) was used to identify the different regulatory mechanisms contributing to various phenotypes of the strains fed with different carbohydrate sources. The present study can be used to identify some key indicators attributed to glucose utilization from multiple dimensions and thus potentially promote efficient selection for future generations that could be more tolerant to dietary carbohydrates.

## 2. Materials and Methods

### 2.1. Ethics Statement

All operations of the present study were complied with the Guidance of the Care and Use of Laboratory Animals in China. This study was approved by the ethics committee of the Institute of Hydrobiology, Chinese Academy of Sciences.

### 2.2. Experimental Diets

Cornstarch (CS), wheat starch (WS), and wheat flour (WF) were used as the carbohydrate sources for the three experimental diets, and the inclusion level was the same as 45%, respectively. Besides that, the three diets had the same composition and levels of energy (19.5 kJ g^−1^ DM), protein (40.0%), and lipids (6.4%). The detailed feed formulations and their chemical analysis are shown in Table S1. Before pelleting, all the ingredients were well ground, passed through 40 mesh (mesh size: 0.25 mm) sieves, evenly mixed, and then made into the pellets as 2 mm diameter to fit the size (3.02 ± 0.82 g) of the experiment fish.

### 2.3. Experimental Fish, Feeding Trial, and Sampling Procedure

There were three strains of gibel carp in the present study, and they were all from the hatchery of the Institute of Hydrobiology, the Chinese Academy of Sciences, Wuhan, Hubei, China. Before the formal trial, a prefeeding period was conducted for 4 weeks to acclimate to the feed and rearing conditions. Then, three strains of the experimental fish were pooled after 24 h starvation. Fish of each strain with the similar weight (3.02 ± 0.82 g) were selected, weighted, and distributed into round fiberglass tanks at the density as 30 fish per tank. Each strain had 9 tanks, respectively, fed by cornstarch (CS) diet, wheat starch (WS) diet, and wheat flour (WF) diet in triplicate, and there were 27 tanks in total (3 strains^∗^ 3 diets^∗^ 3 duplication). The fish were fed to apparent satiation twice a day at 08 : 30 am and 14 : 30 pm and reared in the circulating water system: water volume of each tank was 300 L; diameter of tank was 100 cm; water flow rate was 1.8 L min^−1^; water temperature was 25.5 ± 1.5°C; light intensity was approximately 3 *μ*mol m^−2^ s^−1^ at the water surface; light period was from 08 : 00 am to 20 : 00 pm; water dissolved oxygen was more than 5 mg L^−1^; water ammonia nitrogen was less than 0.5 mg L^−1^; and water residual chloride was less than 0.01 mg L^−1^ (weekly monitored).

The feeding trial lasted for 8 weeks. Then, all fish in each tank were weighed after 24 h of food deprivation, and 4 fish per tank were randomly sampled and frozen at -20°C for whole-body composition analysis. The rest of the fish continued refeeding for one more week; 3 fish/tank were randomly sampled at 6 h after the last meal. They were anesthetized by MS-222 (100 mg L^−1^ tricaine methane sulfonate, Argent Chemical Laboratories Inc., Redmond, WA, USA). After that, blood was collected and then centrifuged at 3000 g, 4°C, 15 min, to obtain the plasma; fresh liver, middle intestine, and muscle (biopsies) of the fish were separated on the ice, immediately frozen in liquid nitrogen, and then kept at -80°C for further analysis.

### 2.4. Chemical Analysis for Diets and Whole-Body Composition

The composition of experiment feed and fish body was analysed according to the methods described by AOAC [24]: dry matter was obtained in an oven at 105°C; crude protein content of feed and fish body was determined by the Kjeldahl method (*N* × 6.25); crude lipid of feed, fish body, and fish liver was extracted with diethyl ether; gross energy of different feed was measured using oxygen bomb calorimeter (6200 Calorimeter, Parr Instrument Company Moline, USA).

### 2.5. Glycogen, Plasma Metabolites, and Amylase Analysis

Liver glycogen and muscle glycogen were determined by the kit from Nanjing Jiancheng Bioengineering Institute, Nanjing, China, according to the amyloglucosidase method. Intestinal amylase was determined by starch-iodine colorimetric method, according to the instructions of the commercial assay kits from Nanjing Jiancheng Bioengineering Institute. Total cholesterol, low-density lipoprotein cholesterol (LDL cholesterol), and high-density lipoprotein cholesterol (HDL cholesterol) were automatically measured by MindrayBS-400 (Mindray Medical International Ltd., Shenzhen, China) according to the instruction, with the glucose oxidase method, GPO-POD method, and cholesterol oxidase method.

### 2.6. mRNA Level Analysis

The transcriptional analysis was conducted on the liver, intestine, and muscle. Total mRNA of the samples was extracted using TRIzol reagent (Invitrogen, Carlsbad, CA, USA). The quality of mRNA was evaluated by spectrophotometry at an absorbance of 260 nm. The integrity of mRNA was visually assessed through agarose gel electrophoresis. Only total RNAs with high RNA ratios (A260/A280 = 1.8 − 2.0) and clear ribosomal bands could be used for further analysis. The obtained total RNAs were reverse transcribed into DNA by Invitrogen TM M-MLV First-Strand Synthesis Kit (Invitrogen, Carlsbad, CA, USA) for further quantification.

Quantitative real-time RT-PCR was next used for measuring the target gene expression by LightCycler 480 II (Roche Diagnostics, Neuilly-sur-Seine, France) using SYBR Green I Master (Roche Diagnostics GmbH, Mannheim, Germany) according to the manufacturer's instructions. As the internal reference gene, *eukaryotic translation elongation factor 1 alpha* (*eef1a*) was used, and the primers of other target genes are shown in Table S2. The present reaction system was 6 *μ*l in total: 3 *μ*l of SYBR Green I Master, 2 *μ*l of the diluted cDNA, 0.52 *μ*l of DNase/RNase/protease-free water, 0.24 *μ*l of forward primer, and 0.24 *μ*l of reverse primer. Quantitative polymerase chain reaction (qPCR) was performed by three steps: initiation at 95°C for 10 min; amplification program (15 s at 95°C, 30 s at the annealing temperature of specific primers and 30 s at 72°C); and cooling at 4°C for 15 min. Melt curve (from 65°C to 95°C with 0.5°C increasement) was regarded as reference to assess the specificity of amplification of primers. The relative quantification of target gene expression was finally calculated by the E-Method, within LightCycler 480 software (version SW 1.5; Roche Diagnostics) as described by Song et al. [27]. PCR efficiency was measured by the slope of a standard curve using serial dilution (10x, 20x, 40x, 80x, 160x, and 320x) of cDNA, and they all ranged between 1.8 and 2.0.

### 2.7. Data Analysis

To provide the overall physiological patterns of different carp strains in response to different carbohydrate sources, 14 parameters regarding growth, feed utilization, and physiological metabolites from 27 samples (data are shown in supplementary Table S3, Table S4, and Table S5) were analysed by self-organizing map (SOM), Spearman's correlation coefficients (*r*), and principal component analysis (PCA). SOMs were devised by Prof. Kohonen [25] based on neural networks trained using competitive learning and can be used to visualize high-dimensional datasets in lower (typically 2) dimensional space. In the present study, SOMs were trained to show the patterns of physiological responses from three carp strains fed three dietary carbohydrate sources. The “kohonen v3.0.10” package was applied to perform the creation and visualization steps for the SOMs [26]. For a descriptive and correlative analysis of the physiological responses of carp to different dietary carbohydrate sources, we used a correlation plot, “ggcorrplot v0.1.3,” in R to determine the associations between the above 14 parameters from 27 samples. The clustering relationships of the physiological index from different carp strains fed different diets or the same strain fed different dietary carbohydrate treatments were described using PCA with the “FactoMineR v2.4” package. The results of PCA were visualized by the “factoextra v1.07” package. A heatmap, a brief visualization method, was used to show the results of gene expression regarding glucose- and lipid-related metabolism in the liver, intestine, and muscle (data are shown in supplementary Table S6, Table S7, and Table S8). The homogenization of the data for relative mRNA levels was applied by the “scale” function, and the clustering and plotting of the data was performed using “pheatmap v1.0.12.”

Furthermore, we performed general statistical analysis for all the above parameters to describe their differences and relationships in a statistical way. After assessing the normality of the distributions by the Shapiro-Wilk test, two-way ANOVA was used to assess the differences between diets, strains, and their interactions, and if there was a statistically significant interaction of diet and strain, a post hoc Tukey test was used to compare between groups. All data were analysed with SPSS 19.0 (SPSS Inc., Chicago, IL, USA). The results are presented as the means ± s.d. (*n* = 3 tanks per group) and are shown in Supplementary Data.

## 3. Results

### 3.1. Growth Performance and Biochemical Indicators in Response to Strains and Carbohydrate Sources

**Figure 1:**
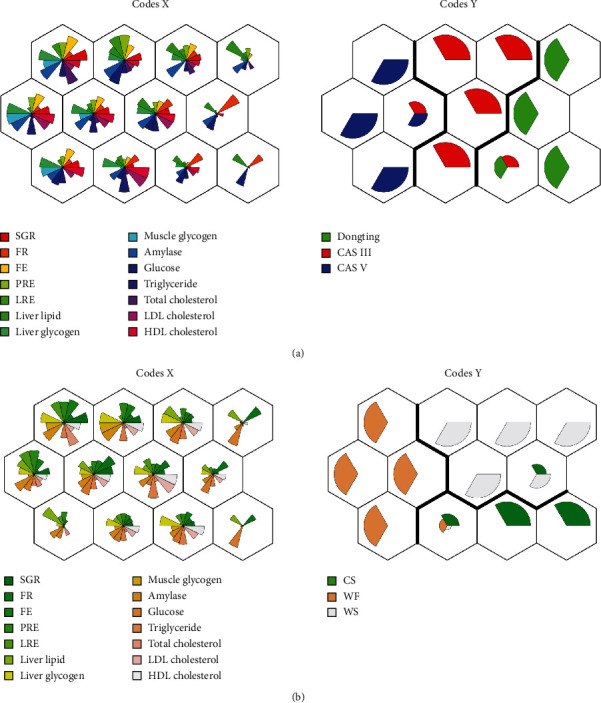
Plots of the codebook vectors of the 4-by-3 mapping for the key variables of the physiological responses from three strains of gibel carp fed different dietary carbohydrate sources using the self-organizing map (SOM). (a) The variables from 27 samples distributed by strain categories. (b) The same variables distributed by carbohydrate source categories. Code *X*: each fan in the neuron represents a variable with different colours, and the weight of a fan corresponds to the magnitude of variable values in a particular dimension. Code *Y*: each treatment position predicted by the characteristic cluster of variables.

The 14 selected variables, including specific growth rate (SGR), feeding rate (FR), feed efficiency (FE), protein retention rate (PRE), lipid retention rate (LRE), liver lipids, liver glycogen, muscle glycogen, intestinal amylase, plasma triglycerides, plasma total cholesterol, plasma LDL cholesterol, and plasma HDL cholesterol, from 27 samples were used to generate two SOMs (Figures 1(a) and 1(b)).  Figure 1(a) shows the distribution of CASV categories on the topological map, and 12 nodes (4-by-3) were obtained. The clustering results of the parameters in the neurons of Code *X* are represented by the fans with different colours, while the corresponding characteristics are shown in the same position in those of Code *Y*. It can be seen that the distribution of samples from the three strains in each neuron was very different: CASV was mainly distributed on the left side, CASIII was distributed in the middle, and Dongting was distributed on the right.

The contribution of the 14 parameters (the relative weight of the fan) to neuron nodes can explain the characteristics of the three strains in response to high carbohydrate intake (45%). For instance, SGR, FE, PRE, and LRE had relatively larger weights in CASV, followed by CASIII and then Dongting. This result indicated that CASV had higher growth and feed utilization than CASIII, and these values were higher than those of Dongting. Liver lipid and plasma glucose seemed higher in Dongting, which indicated the poor regulation of postprandial glucose in Dongting and thus resulted in liver fat accumulation. Muscle glycogen was higher in CASV than in CASIII and Dongting. Total cholesterol, LDL cholesterol, and HDL cholesterol were all higher in CASV and CASIII than in Dongting. Moreover, the distribution of diet categories was characterized as CS > WF > WS, as shown in Figure 1(b), and the cluster results showed that WF was characterized by a higher SGR, FE, PRE, LRE, liver lipid, and amylase. CS was characterized by a higher plasma glucose. WS was characterized by a higher total cholesterol, LDL cholesterol, and HDL cholesterol.

**Figure 2:**
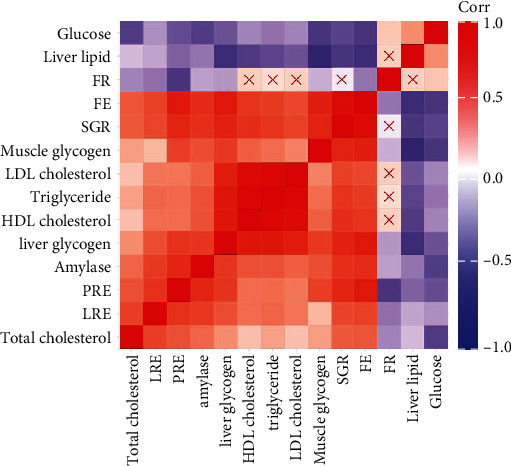
Correlation plot for the associations between 14 parameters reflecting the physiological responses of three gibel carp strains fed different dietary carbohydrate sources. The colours represent the degree of pairwise correlation regarding Spearman's correlation coefficient (*r*). Red indicates a significantly positive correlation, blue indicates a significantly negative correlation, and *X* indicates no significant correlation.

We performed Spearman's correlation analysis on the above 14 parameters from 27 samples (Figure 2). The blue colour represents a negative correlation, while the red colour represents a positive correlation. *X* means that the correlation was not significant between the two parameters. Figure 2 shows that total cholesterol, LRE, PRE, amylase, liver glycogen, HDL cholesterol, triglyceride, LDL cholesterol, muscle glycogen, SGR, and FE were significantly positively correlated with each other and significantly negatively correlated with plasma glucose and liver lipids. Plasma glucose and liver lipids had a significant positive correlation. FR was significantly positively correlated with glucose and significantly negatively correlated with FE, muscle glycogen, liver glycogen, amylase, PRE, LRE, and total cholesterol. FR had no significant correlation with SGR, LDL cholesterol, triglyceride, or HDL cholesterol.

**Figure 3:**
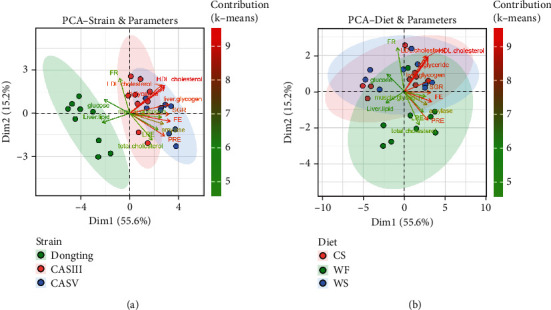
Principal component analysis (PCA) biplot of physiological responses from three gibel carp strains fed different dietary carbohydrate sources. (a) The PCA performed at the strain level. (b) The PCA performed at the carbohydrate source level. Arrows indicate the direction of increasing values for each variable. Colour coding indicates cluster membership determined by *K*-means clustering based on their scores on the main PCA dimensions.

Next, the above 14 parameters from 27 samples were subjected to principal component analysis (PCA) at both the strain level (Figure 3(a)) and diet level (Figure 3(b)). The results showed that (1) the two axes, Dim1 and Dim2, explained 55.6% and 15.2% of the variation in all variables, respectively, and that (2) plasma glucose had a positive correlation with FR and liver lipids but a negative correlation with most parameters, such as total cholesterol, LRE, PRE, amylase, liver glycogen, HDL cholesterol, triglyceride, LDL cholesterol, muscle glycogen, SGR, and FE. LDL cholesterol, triglyceride, and HDL cholesterol were significantly positively correlated with each other, which was consistent with Spearman's analysis. Additionally, (3) the characteristics of CASIII and CASV in response to the carbohydrate diet were relatively similar, as the plots from CASIII and CASV clustered together, and the graphs of CASIII and CASV overlapped. However, these strains acted differently from Dongting, as the plots from Dongting were scattered in different directions compared to CASIII and CASV, and the graph of Dongting was totally separated from the graphs of CASIII and CASV. Moreover, the results of PCA regarding carbohydrate sources (Figure 3(b)) showed that the characteristics of the fish fed CS and WS were relatively similar, but they were different from the characteristics of the fish fed WF.

**Figure 4:**
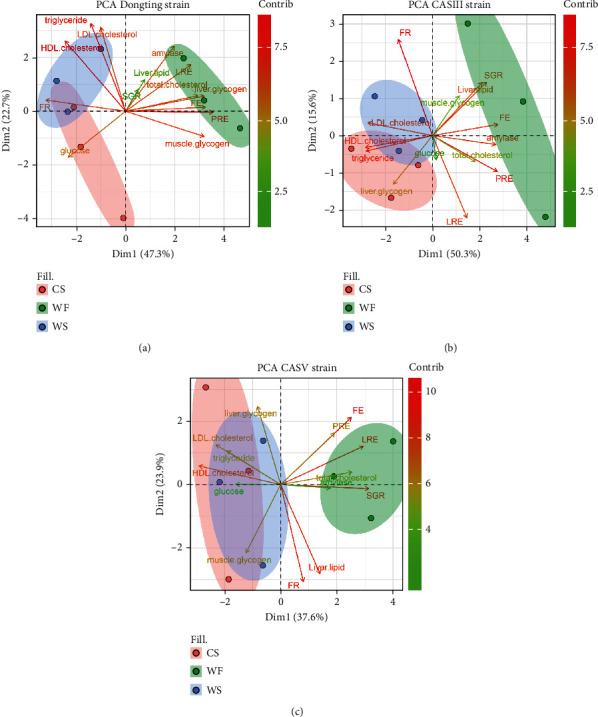
Principal component analysis (PCA) biplot of physiological responses from each strain of gibel carp fed three dietary carbohydrate sources. (a) The physiological responses of Dongting fed CS, WF, and WS. (b) The physiological responses of CASIII fed CS, WF, and WS. (c) The physiological responses of CASV fed CS, WF, and WS. Arrows indicate the direction of increasing values for each variable. Colour coding indicates cluster membership determined by *K*-means clustering based on their scores on the main PCA dimensions.

To further study the diversity in carbohydrate utilization by strains, we conducted PCA on the parameters for each strain fed three carbohydrate sources.  Figure 4(a) refers to Dongting fed CS, WF, and WS; Figure 4(b) refers to CASIII fed CS, WF, and WS; and Figure 4(c) refers to CASV fed CS, WF, and WS. All graphs have two axes (Dim1 and Dim2), accounting for 70.0%, 65.9%, and 61.5% of the variance in the data, reflecting the high quality of the cluster.  Figure 4(a) shows that the use of WF was most different from that of CS and WS for Dongting. Figure 4(b) shows that CASIII had some similarities in the use of CS and WS but had different performance when fed WF. Figure 4(c) shows that CASV had a very similar use of CS and WS, represented by the graphs of CS and WS largely overlapping, and the utilization of WF was markedly different from that of CS and WS.

### 3.2. Regulation of Genes Involved in Glucose and Lipid Metabolism in Three Carp Strains Fed Different Carbohydrate Sources

**Figure 5:**
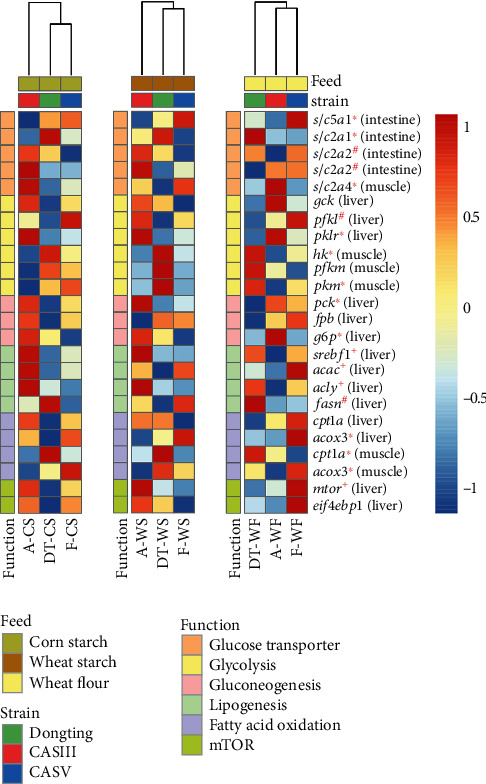
Heatmap illustrating the main patterns of gene expression involved in glucose and lipid metabolism within three strains fed different carbohydrate sources. Similarities between the groups, as shown by hierarchical clustering, can be seen above the heatmap with bootstraps. The intensity of the colour indicates the expression level: red, upregulated expression; blue, downregulated expression. Superscripts are labelled if there are significant differences in gene expression according to the statistical analysis: ^∗^ indicates a significant difference in gene expression between strains; + indicates a significant difference in gene expression between carbohydrate sources; and # indicates a significant interaction of gene expression between strains and carbohydrate sources.

The expression of genes related to glucose and lipid metabolism is displayed in a heatmap (Figure 5). If the gene is upregulated, then the corresponding box is red, and the darker red colour, the stronger the upregulation. Otherwise, if the gene is downregulated, the corresponding box is blue, and the darker blue colour, the stronger the downregulation. The cluster of all genes revealed that the characteristics of gene expression were close between Dongting and CASV, and they were different with CASIII when the fish were fed CS and WS. Although CASIII and CASV had similar gene expression profiles, they exhibited different transcriptional characteristics with Dongting when fed WF.

### 3.3. Supplementary Result

All the results were also performed using statistical analysis, which was described in Supplementary Result.

## 4. Discussion

The carbohydrate content in the present diets was set based on the results of the previous studies in which gibel carp was found to tolerate a high carbohydrate level of 45% in feed [5, 27]. Here, we focused on the responses of gibel carp genotypes to different carbohydrate sources to investigate the characteristics of growth, feed utilization, and the regulation of postprandial glycaemia under high carbohydrate intake within both genotypes and carbohydrate sources. We aimed to understand the patterns of physiological responses to carbohydrate diets for fish and determine the metabolic traits of efficient carbohydrate use between genotypes to provide scientific support for the subsequent selection of gibel carp strains.

### 4.1. Postprandial Hyperglycaemia Caused by High Carbohydrate Intake Was Closely Related to Liver Fat and Affected the Growth of Gibel Carp

Lipid biosynthesis is highly correlated with dietary carbohydrates [28]. Previous studies have shown that rainbow trout [27] and gibel carp [17, 18] increase lipid synthesis in response to high carbohydrate intake to alleviate the plasma glucose load. A similar effect was observed here, and a significant positive correlation between plasma glucose and liver lipids was confirmed by Spearman's correlation analysis in the present study. Alarmingly, postprandial plasma glucose was negatively correlated with SGR, FE, PRE, and LRE. These results explained a phenomenon that commonly occurs in cultured fish in which a carbohydrate-enriched diet causes fatty liver in fish, thus increasing mortality and decreasing their growth performance [29]. Excess glucose derived from feeding always results in either glycogen synthesis [4] or an increase in triglycerides and cholesterols [30]. In contrast, plasma glucose in the present study was also negatively correlated with metabolites such as plasma triglycerides, liver glycogen, muscle glycogen, and cholesterols (HDL, LDL, and total cholesterol). This may be due to the sampling time; plasma glucose rose rapidly within 0-3 h after the meal, and glycogen and lipid metabolites increased accordingly. At 6 h after the last meal, plasma glucose had decreased, while glycogen and lipid metabolites were still at high levels, which may be the reason why they presented a negative correlation in the present study.

### 4.2. Genetic Variability in Growth Performance and Intermediary Metabolism

The cluster of growth and physiological responses revealed that the selected strains CASV and CASIII were very similar, and they were different from the wild strain Dongting. Dongting had relatively lower growth and feed utilization compared to CASIII and CASV and had lower SGR, FE, PRE, and LRE than the other two strains, whereas CASV showed relatively better performance. This was consistent with the previous studies showing that CASIII and CASV had higher bioavailability of dietary nutrients by accelerating the mTOR pathway and enhancing protein synthesis, thus contributing to better growth and feed efficiency [31]. The present study further confirmed the superior ability of CASV to use dietary carbohydrates, which could be explained by the following three observations: (1) the enhanced activity of amylase in CASV may be associated with the utilization of carbohydrates; (2) higher PRE, LRE, and FE strongly reflected more protein sparing by carbohydrates in CASV; and (3) enhanced expression of *mtor* and *eif4ebp1* indicated an accelerated mTOR pathway in CASV-fed WF. Dongting showed high potential for lipid synthesis, especially when stimulated by high carbohydrate levels (Song et al., unpublished). The liver lipids in the present study were in accordance with the previous finding that they were higher in Dongting than in CASV. Diverse metabolic responses to high carbohydrate intake between strains for coping with postprandial glycaemia were also observed in the present study. First, glucose was stored more often as glycogen in CASV than in CASIII and Dongting. Second, CASIII and CASV highly induced plasma triglyceride and cholesterol (HDL, LDL, and total cholesterol) synthesis, which was abundantly clear from many models showing that conversion from glucose to lipid was an efficient pathway to control plasma glucose [28].

### 4.3. Wheat Flour Was Associated with Higher Feed Efficiency and Affected Lipid Storage in Gibel Carp

The clustering based on carbohydrate sources showed very high similarity when fish were fed cornstarch (CS) and wheat starch (WS), while the fish performance was different when fed wheat flour (WF). Compared with those fed CS and WS, fish fed WF had a higher FE, PRE, and LRE. This may be due to the higher digestibility of WF; the apparent digestibility coefficients of dry matter and crude protein in wheat flour could be up to 100% [32]. Accordingly, amylase activity was more highly induced when the fish were fed WF, which further contributed to the efficient use of WF. In the present study, liver lipids were also increased in fish fed WF. The above results were in line with the observation for European eel (*Anguilla anguilla* L.) that the percent of fat, protein retention, and energy retention were all higher for eels fed wheat meal or bread meal than for eels fed other carbohydrate sources [33]. The authors considered that the positive effects on fat retention, growth, and feed utilization impacted the fatty acid composition of eels fed wheat diets. The lipid metabolites in the present study seem to confirm this speculation. Plasma triglycerides and cholesterols (LDL, HDL, and total cholesterol) were significantly affected by carbohydrate sources, and they were higher in the WF group. Moreover, the genes *srebf1*, *acac*, *acly*, and *fasn* involved in lipid synthesis were all upregulated by feeding WF. Hence, we can infer that carbohydrate sources may affect fat metabolism from *de novo* lipogenesis and that fatty acid composition may affect phenotypical fat synthesis and distribution in fish, but the relevance of carbohydrate sources to lipid and cholesterol metabolism remains to be further investigated.

### 4.4. Diverse Transcriptional Regulation in Three Gibel Carp Strains in Response to Different Carbohydrate Sources

A previous study found a more efficient regulation of postprandial glycaemia in Nile tilapia *(Oreochromis niloticus*), in which glycolysis and lipogenesis were highly accelerated, while gluconeogenesis was decelerated under high carbohydrate intake [34]. In the present study, target genes involved in glucose transportation, glycolysis, gluconeogenesis and lipogenesis, and fatty acid oxidation in major tissues were determined to evaluate the contribution of these metabolic pathways to glucose homeostasis in gibel carp fed different carbohydrate sources. Interestingly, transcriptional characteristics were similar between CASV and Dongting fed CS and WS, while CASV and CASIII showed similarity in the use of WF. The characteristics of gene expression are described according to their functions as follows: *slc5a1* is regarded as a glucose sensor in the trout intestinal tract [35]. When fish receive a glucose stimulus, upregulated *slc5a1* (previously named *sglt1*) mRNA is found in fish [36, 37], and this upregulation also increases the levels of glucose transporter 2 (*glut2*, now named *slc2a2*, used in the present study) in the basolateral membrane of the intestine in rainbow trout [38]. Here, we found different expression patterns for *slc5a1* and *gluts* in the intestine, liver, and muscle among three gibel carp strains. Generally, glucose transporters were affected more by gibel carp genotypes than by carbohydrate sources. In the present study, *slc5a1* was more highly expressed and induced *slc2a2* in the intestine and *slc2a4* in the muscle of CASV. Intestinal *slc2a1* expression did not match that of *slc5a1* and *slc2a2,* which was higher in Dongting. There was a strong interaction of hepatic *slc2a2* expression between fish genotypes and carbohydrate sources. CASIII had relatively higher *slc2a2* mRNA levels than Dongting and CASV fed CS, and there was no significant difference in *slc2a2* mRNA levels among the three strains when fed WS and WF.

Various regulatory effects of genotypes on glycolysis and gluconeogenesis were also observed. CASIII had a higher mRNA level of *pklr*, which encodes the last glycolytic enzyme in the liver; Dongting upregulated glycolysis in the muscle by inducing *hk* and *pkm* expression; and CASV showed lower gene expression of *g6p*, which was reported to be a critical factor for inducing gluconeogenesis [39]. Some differences in *pklr* are related to hepatic glycolysis, and *pck* is involved in hepatic gluconeogenesis. Their mRNA levels were relatively higher in fish fed WF. However, the present data were not sufficient to reveal the systematic effects of carbohydrate sources on glycolytic and gluconeogenetic metabolism; therefore, more studies are needed in the future.

The lipogenic genes *srebf1*, *acac*, *acly*, and fasn were upregulated by WF, and they were slightly affected by genotype. However, the changes in the mRNA levels of the genes involved in fatty acid oxidation showed genotype-related regulation. CASV showed upregulated *acox3* in the liver and muscle, and Dongting had relatively higher mRNA levels of *cpt1a* and *acox3* in the muscle. The muscle is the largest tissue of the fish body and plays a major role in the use of nutrients. Previous studies revealed a low capacity for glucose utilization in fish muscle because of the low efficiency of glucose uptake by muscle though glucose transporters and poor activation of enzymes involved in glycolysis when fed a carbohydrate-enriched diet [40]. This study provides new insight into lipid synthesis and fatty acid oxidation in muscle, which may also be associated with glucose homeostasis in fish.

## 5. Conclusion

The present study evaluated the relationship of carbohydrate sources and glucose metabolism from different dimensions and confirmed that wheat flour was the preferable carbohydrate source for gibel carp owing to the observed better growth performance and feed utilization. The results also demonstrated the existence of genetic variability in growth, feed utilization, and postprandial plasma glucose regulation between the wild-type strain and artificially selected strains of gibel carp. In general, selected CASV had higher growth performance, and CASV and CASIII showed relatively better regulation of glucose-related metabolites, while Dongting exhibited postprandial glycaemia, poor growth, and feed utilization. In addition, CASIII induced hepatic glycolysis, CASV restrained hepatic gluconeogenesis, and Dongting activated glucose and lipid metabolism in the muscle. The present study helps increase our understanding of carbohydrate use in fish and may assist in promising genetic selection to improve glucose homeostasis in fish.

## Data Availability

The data that support the findings of this study are available from the corresponding author upon reasonable request.

## Supplementary Materials

Supplementary Materials To compare the differences in detail and make the comparison clear, we also performed statistical analysis on all the results, which was shown in the Supplementary Data. Accordingly, the statistical results were described and showed in the Supplementary Result.
