# Supplementary material for: Data Mining Evidences Variabilities in Glucose and Lipid Metabolism among Fish Strains: A Case Study on Three Genotypes of Gibel Carp Fed by Different Carbohydrate Sources
Source: Aquac Nutr. 2023 Feb 2;2023:7589827. doi: 10.1155/2023/7589827 (PMC9973145; doi:10.1155/2023/7589827)
Supplement: Supplementary Materials — To compare the differences in detail and make the comparison clear, we also performed statistical analysis on all the results, which was shown in the Supplementary Data. Accordingly, the statistical results were described and showed in the Supplementary Result. [file 7589827.f1.zip › Supplementary data (1).docx]

Table S1. Formulation and chemical composition of diets with different carbohydrate sources.

|  | CS | WS | WF |
| --- | --- | --- | --- |
| Ingredients (% DM) |  |  |  |
| White fish meal^1^ | 20.00 | 20.00 | 20.00 |
| Casein^2^ | 23.00 | 23.00 | 16.00 |
| Fish oil^3^ | 3.25 | 3.25 | 3.25 |
| Soybean oil^4^ | 3.25 | 3.25 | 3.25 |
| Corn starch^5^ | 45.00 | - | - |
| Wheat starch | - | 45.00 | - |
| Wheat flour | - | - | 45.00 |
| Choline chloride | 0.11 | 0.11 | 0.11 |
| Cellulose | 0 | 0 | 7.00 |
| Vitamin premix^6^ | 0.39 | 0.39 | 0.39 |
| Mineral premix^7^ | 5.00 | 5.00 | 5.00 |
| Chemical composition | | |  |
| Crude protein (% DM) | 42.09 | 39.43 | 40.70 |
| Crude lipid (% DM) | 6.40 | 6.43 | 6.47 |
| Gross energy (kJ g^-1^ DM) | 19.7 | 19.2 | 19.5 |

CS, Corn starch diet; WS, Wheat starch diet; WF, Wheat flour diet; DM, dry matter

^1^ American Seafood Company, Seattle, Washington, USA

^2^ Sigma Chemical Co., St. Louis, MO, USA

^3^ Anchovy oil from Peru, purchased from Coland Feed, Wuhan, Hubei, China

^4^ Yihai Kerry Y Co., Ltd., purchased from Wuhan, Hubei, China

^5^ Wuhan Coland Feed Co., Ltd., Wuhan, Hubei, China

^6^ Vitamin premix (mg kg^-1^ diet): thiamin, 20; riboflavin, 20; pyridoxine, 20; cyanocobalamine, 0.02; folic acid, 5; calcium pantothenate, 50; inositol, 100; niacin, 100; biotin, 0.1; Starch, 645.2; ascorbic acid, 100; Vitamin A, 110; Vitamin D, 20; Vitamin E, 50; Vitamin K, 10

^7^ Mineral premix (mg kg^-1^ diet): NaCl, 500; MgSO_4_-7H_2_O, 4575.0; NaH_2_PO_4_-2H_2_O, 12500.0; KH_2_PO_4_, 16000.0; Ca(H_2_PO_4_)_2_-H_2_O, 6850.0; FeSO_4_, 1250.0; C_6_H_10_CaO_6_-5H_2_O, 1750.0; ZnSO_4_-7H_2_O, 111.0; MnSO_4_-4H_2_O, 61.4; CuSO_4_-5H_2_O, 15.5; CoSO_4_-6H_2_O, 19.02; KI, 178.33; Starch, 6253.33

| Table S2. Primer sequences and accession numbers for qPCR analysis. | | | |
| --- | --- | --- | --- |
| Gene  name | Forward  primer (5'-3') | Reverse  primer (5'-3') | GenBank accession  number |
| Reference gene | | | |
| *eef1α* | GTTGGAGTCAACAAGATGGACTCCAC | CTTCCATCCCTTGAACCAGCCCAT | AB056104 |
| mTOR | | | |
| *mtor* | TTGATGGCACGGTGTTTCCTAA | GCCCTGGTTCTGGTGCTTGTAG | - |
| *eif4ebp1*: | CCCCTGCTGCCTCCCTAACATT | TCAGTCATCGCCGCCTTTG | - |
| Glucose transporters | | | |
| *slc5a1* | CGTGCTGTCCCTGTTTCTC | GACCACCTGTTACTGTAT | MH539755 |
| *slc2a1* | CACTGTTGTGTCTCTGTTTGTGG | AGATTGCTATGATGCTAATGTAGGACATC | KY328741 |
| *slc2a2* | CTCGTGGATGAGCTACCTCAGCAT | CCCTGACTGAAGATCTCCGCCA | KX898504 |
| *slc2a4* | CACTCTCTGTGGCCATCTTCTCCAT | TCACCAACATACATGGGCACCAATCCT | KY328742 |
| Glycolysis | | | |
| *gck* | GAGGAGATGCGTAAGGTGGAGCT | TTCTCATACAGCTGATGTCCAGGGTT | KX898498 |
| *hk* | TATGAGAAGATGATCAGCGGGATGTACC | ATCACACGTGCTGCTGATCAAGCCCA | KX898499 |
| *pfkl* | ACACCGGATGCCGCAGAAGCA | TCGATCTCTCCGGTCACATACTCG | KX898500 |
| *pfkm* | TAGCTATCATGAACGTTGGAGCTCC | CTTTGCCTGTCCAGCCTCCAACA | KX898501 |
| *pklr* | GCATCTGTGTCTGCTGGACATCGA | TGAGAGCCGTGAGAGAAGTTCAGTC | KX898502 |
| *pkm* | ATCATGGAGGCCAGCGATGGCAT | GGCTTCTTGATCATGCTCTCCAACAT | KX898503 |
| Gluconeogenesis | | | |
| *pck* | AGACAAACCCTCATGCCATGGCAAC | GGGTCTATGATGGGGCACTGG | KX898506 |
| *fpb* | CACAAATGTTACAGGTGACCAGGTGAA | AATGTTTGAAGAGCCATCCAGAGGGT | GU593002 |
| *g6p* | CCTTACTGGTGGGTCCATGAGACT | TGGGCCGGTCTCACAGGTCAT | KX898505 |
| Lipid synthesis | | | |
| *srebf1* | GGCCCTCTACTGCGTGGCACA | ACCACCATTTGGAGTGAGGGTCAC | KX898507 |
| *acly* | AGTTTGGCCACGCTGGAGCTTGT | CCCAGCTCATCGAAGCTCTTGG | KX898508 |
| *acac* | GAGCTGTCTATCAGAGGAGACTTCA | GACGCTCGGCCTGCATCTTCT | KF499584 |
| *fasn* | CCACACCATGGACCCACAGCT | CTGGGTCTTTACTGAAGGCCTCT | KF511494 |
| Fatty acid oxidation | | | |
| *cpt1a* | GAAGCTCATCAGGCTGTGGCCTT | TTCCAGGAGTGAAGTCCGGAGAG | KX898509 |
| *acox3* | TGTGGAGGACACGGTTACCTTGC | AGTTGCTGGTCTGCTGCAGAAGG | KX898510 |

Reference gene: *eef1α*, *eukaryotic translation elongation factor 1 alpha*.

mTOR: *mtor*: *mammalian target of rapamycin*; *eif4ebp1*: *eukaryotic translation initiation factor 4E-binding protein 1*.

Glucose transporters: *slc5a1*, *solute carrier family 5 member 1*; *slc2a1*, *solute carrier family 2 member 1*; *slc2a2*, *solute carrier family 2 member 2*; *slc2a4*, *solute carrier family 2 member 4*

Glycolysis: *gck*, *glucokinase*; *hk*, *hexokinase*; *pfkl*, *6-phosphofructokinase*, *liver type*; *pfkm*, *6-phosphofructokinase*, *muscle type*; *pklr*, *pyruvate kinase*, *liver type*. *pkm*, *pyruvate kinase*, *muscle type*

Gluconeogenesis: *pck*, *phosphoenolpyruvate carboxykinase*; *fbp*, *fructose 1,6-bisphosphatase*; *g6p*, *glucose-6-phosphatase*

Lipid synthesis: *srebf1*, *sterol regulatory element binding transcription factor 1*; *acly*, *ATP citrate lyase*; *acac*, *acetyl-CoA carboxylase*; *fasn*, *fatty acid synthase*

Fatty acid oxidation: *cpt1a*, *carnitine palmitoyl transferase 1 isoform alpha*; *acox3*, *acyl-CoA oxidase 3*

Table S3. Growth performance in three strains of gibel carp fed CS diet, WS diet and WF diet. Data were presented as mean±s.d. (n=3 tanks), statistical differences were evaluated by Two-way *ANOVA* (*p*<0.05, values in bold). In case of interaction, mean values were evaluated by One-way *ANOVA* (*p*<0.05, values in bold) followed by a *post-hoc* Tukey’s test. ^A, B^ Different capital letters indicate significant differences between strains (*p*<0.05). ^a, b^ Different lowercase letters indicate significant differences between diets (*p*<0.05).

|  | CS | | | WS | | | WF | | | | *P*-value | | |
| --- | --- | --- | --- | --- | --- | --- | --- | --- | --- | --- | --- | --- | --- |
|  | Dongting | CASIII | CASV | Dongting | CASIII | CASV | Dongting | CASIII | CASV | Diet | | Strain | Diet*Strain |
| Inital body weight | 1.9±0.2^a^ | 3.2±0.1^b^ | 3.9±0.2^c^ | 1.9±0.1^a^ | 3.3±0.1^b^ | 3.8±0.1^c^ | 2.0±0.2^a^ | 3.3±0.2^b^ | 3.8±0.0^c^ | 0.928 | | **≤0.001** | 0.667 |
| Final body weight | 4.6±0.4^a^ | 10.1±0.5^b^ | 17.5±1.0^d^ | 4.6±0.3^a^ | 11.7±0.4^b^ | 17.7±0.9^d^ | 4.8±0.8^a^ | 14.9±1.0^c^ | 22.9±0.8^e^ | **≤0.001** | | **≤0.001** | **≤0.001** |
| FR (% BW d^-1^) | 4.8±0.4 | 4.6±0.4 | 4.4±0.3 | 5.1±0.3 | 4.9±0.3 | 4.6±0.2 | 4.1±0.4 | 4.7±0.6 | 4.6±0.2 | 0.050 | | 0.457 | 0.098 |
| SGR (% d^-1^) | 1.6±0.1^a^ | 2.1±0.1^b^ | 2.7±0.1^c^ | 1.7±0.1^a^ | 2.3±0.1^b^ | 2.8±0.1^c^ | 1.7±0.1^a^ | 2.7±0.2^c^ | 3.2±0.1^d^ | **≤0.001** | | **≤0.001** | **0.005** |
| FE (%) | 31.2±1.7^Aa^ | 41.6±1.2^Ba^ | 51.4±2.7^Ca^ | 30.9±1.3^Aa^ | 42.2±1.2^Ba^ | 50.4±1.8^Ca^ | 36.8±0.1^Ab^ | 46.2±1.4^Bb^ | 55.8±1.6^Cb^ | **≤0.001** | | **≤0.001** | 0.894 |
| PRE (%) | 15.5±1.5^Aa^ | 18.1±1.0^ABa^ | 20.2±1.5^Ba^ | 15.6±0.7^Aab^ | 18.9±1.3^ABab^ | 21.5±0.4^Bab^ | 19.7±1.9^Ab^ | 21.7±3.0^ABb^ | 22.1±1.1^Bb^ | **0.005** | | **≤0.001** | 0.506 |
| LRE (%) | 48.6±3.2^Aa^ | 61.1±1.5^ABa^ | 58.3±5.2^Ba^ | 50.7±2.9^Aa^ | 59.3±5.3^Aa^ | 55.5±3.7^Ba^ | 57.6±2.4^Ab^ | 63.8±9.7^ABb^ | 66.9±2.6^Bb^ | **0.010** | | **0.003** | 0.637 |

CS, corn starch diet; WS, wheat starch diet; WF, wheat flour diet

Feeding rate (FR, %BW d^-1^) =100 × (feed intake in dry matter, g) / [days × (initial body weight, g + final body weight, g)/2]

Specific growth rate (SGR, % d^-1^) = 100 × [Ln (final body weight, g) - Ln (initial body weight, g)]/ days

Feed efficiency (FE, %) = 100 × (wet weight gain, g) / (feed intake in dry matter, g)

Protein retention efficiency (PRE, %) =100 × (final body protein – initial body protein) / total protein intake

Lipid retention efficiency (LRE, %) = 100 × (final body lipid – initial body lipid) / total lipid intake

Table S4. Tissue composition and intestinal amylase in three strains of gibel carp fed CS diet, WS diet and WF diet. Data were presented as mean±s.d. (n=3 tanks), statistical differences were evaluated by Two-way *ANOVA* (*p*<0.05, values in bold). In case of interaction, mean values were evaluated by One-way *ANOVA* (*p*<0.05, values in bold) followed by a *post-hoc* Tukey’s test. ^A, B^ Different capital letters indicate significant differences between strains (*p*<0.05). ^a, b^ Different lowercase letters indicate significant differences between diets (*p*<0.05).

|  | CS | | | WS | | | WF | | | *P*-value | | |
| --- | --- | --- | --- | --- | --- | --- | --- | --- | --- | --- | --- | --- |
|  | Dongting | CASIII | CASV | Dongting | CASIII | CASV | Dongting | CASIII | CASV | Diet | Strain | Diet*Strain |
| Liver lipid (%) | 4.5±0.7^Ba^ | 5.0±0.0^Ba^ | 3.0±1.0^Aa^ | 6.7±1.2^Bb^ | 6.7±1.2^Bb^ | 3.3±0.6^Ab^ | 8.7±3.5^Bc^ | 7.3±0.6^Bc^ | 3.7±0.6^Ac^ | **0.016** | **≤0.001** | 0.407 |
| Liver glycogen (mg/g) | 54.3±8.3^a^ | 115.2±21.4^d^ | 116.3±6.9^d^ | 61.1±5.3^a^ | 106.8±6.4^cd^ | 117.0±10.6^d^ | 73.3±5.1^ab^ | 93.1±17.5^bc^ | 115.4±10.8^d^ | 0.939 | **≤0.001** | **0.003** |
| Muscle glycogen (mg/g) | 4.4±0.9^A^ | 6.0±1.9^A^ | 15.2±2.9^B^ | 3.6±0.6^A^ | 5.1±1.8^A^ | 17.6±3.6^B^ | 6.4±1.7^A^ | 8.1±2.3^A^ | 14.3±2.7^B^ | 0.570 | **≤0.001** | 0.153 |
| Amylase (U/g protein) | 334.4±16.8^Aa^ | 436.2±6.5^Ba^ | 437.9±16.4^Ba^ | 395.0±35.2^Aab^ | 449.8±15.0^Bab^ | 495.5±27.4^Bab^ | 443.3±70.4^Ab^ | 471.4±22.3^Bb^ | 494.7±26.6^Bb^ | **≤0.001** | **≤0.001** | 0.283 |

CS, corn starch diet; WS, wheat starch diet; WF, wheat flour diet

DT strain, gibel carp Dongting; A strain, gibel carp CASIII; F strain, gibel carp CASV

Table S5. Plasma metabolites in three strains of gibel carp fed CS diet, WS diet and WF diet at 6h after the last meal. Data were presented as mean±s.d. (n=3 tanks), statistical differences were evaluated by Two-way *ANOVA* (*p*<0.05, values in bold). In case of interaction, mean values were evaluated by One-way *ANOVA* (*p*<0.05, values in bold) followed by a *post-hoc* Tukey’s test. ^A, B^ Different capital letters indicate significant differences between strains (*p*<0.05). ^a, b^ Different lowercase letters indicate significant differences between diets (*p*<0.05).

| Plasma metabolites  (mmol L^‑1^) | CS | | | WS | | | WF | | | | *P*-value | | |
| --- | --- | --- | --- | --- | --- | --- | --- | --- | --- | --- | --- | --- | --- |
|  | Dongting | CASIII | CASV | Dongting | CASIII | CASV | Dongting | CASIII | CASV | Diet | | Strain | Diet*Strain |
| Glucose | 6.8±0.9^b^ | 6.3±0.9^ab^ | 6.3±1.4^ab^ | 7.0±0.9^b^ | 6.0±0.7^ab^ | 4.8±0.5^ab^ | 5.8±0.3^ab^ | 7.3±0.8^b^ | 4.4±0.4^a^ | 0.147 | | **0.005** | **0.046** |
| Triglyceride | 2.8±0.3^a^ | 4.1±0.9^a^ | 3.0±0.4^a^ | 2.9±0.4^a^ | 3.6±0.8^a^ | 3.7±0.9^a^ | 4.4±1.0^ab^ | 4.6±0.8^ab^ | 6.9±1.7^b^ | **≤0.001** | | **0.035** | **0.046** |
| Total cholesterol | 4.7±06^Aab^ | 9.9±0.4^Bab^ | 8.8±0.9^Bab^ | 5.6±0.2^Ab^ | 10.1±1.1^Bb^ | 9.3±0.8^Bb^ | 5.0±0.3^Aa^ | 7.9±0.5^Ba^ | 8.4±1.0^Ba^ | **0.007** | | **≤0.001** | 0.110 |
| LDL cholesterol | 1.0±0.2^Aab^ | 2.6±0.2^Bab^ | 2.2±0.5^Bab^ | 1.1±0.0^Ab^ | 2.8±0.3^Bb^ | 2.4±0.1^Bb^ | 1.1±0.1^Aa^ | 2.0±0.3^Ba^ | 1.9±0.3^Ba^ | **0.004** | | **≤0.001** | 0.105 |
| HDL cholesterol | 2.3±0.2^Aab^ | 4.2±0.3^Bab^ | 4.2±0.2^Bab^ | 2.7±0.1^Ab^ | 4.3±0.3^Bb^ | 4.1±0.3^Bb^ | 2.2±0.2^Aa^ | 3.6±0.2^Ba^ | 3.7±0.3^Ba^ | **≤0.001** | | **≤0.001** | 0.207 |

CS, corn starch diet; WS, wheat starch diet; WF, wheat flour diet

LDL cholesterol: low-density lipoprotein cholesterol; HDL cholesterol: high-density lipoprotein cholesterol

Table S6. mRNA levels of selected genes in the intestine of three strains of gibel carp fed CS diet, WS diet and WF diet at 6h after the last meal. Data were presented as mean±s.d. (n=3 tanks), statistical differences were evaluated by Two-way *ANOVA* (*p*<0.05, values in bold). In case of interaction, mean values were evaluated by One-way *ANOVA* (*p*<0.05, values in bold) followed by a *post-hoc* Tukey’s test. ^A, B^ Different capital letters indicate significant differences between strains (*p*<0.05). ^a, b^ Different lowercase letters indicate significant differences between diets (*p*<0.05).

| Genes expression | CS | | | WS | | | WF | | | *P*-value | | |
| --- | --- | --- | --- | --- | --- | --- | --- | --- | --- | --- | --- | --- |
|  | Dongting | CASIII | CASV | Dongting | CASIII | CASV | Dongting | CASIII | CASV | Diet | Strain | Diet*Strain |
| Glucose transporters | | | | | | | | | | | | |
| *slc5a1* | 0.99±0.06^AB^ | 0.68±0.11^A^ | 1.03±0.07^B^ | 0.87±0.07^AB^ | 0.48±0.03^A^ | 1.23±0.04^B^ | 1.03±0.08^AB^ | 0.90±0.03^A^ | 1.34±0.06^B^ | 0.175 | **≤0.001** | 0.633 |
| *slc2a1* | 1.15±0.13^B^ | 0.59±0.07^A^ | 0.76±0.05^A^ | 1.10±0.23^B^ | 0.95±0.22^A^ | 0.72±0.17^A^ | 1.34±0.47^B^ | 0.84±0.14^A^ | 0.81±0.28^A^ | 0.109 | **≤0.001** | 0.140 |
| *slc2a2* | 1.13±0.14^ab^ | 1.15±0.48^ab^ | 0.90±0.19^ab^ | 1.05±0.24^ab^ | 1.31±0.44^ab^ | 0.72±0.18^a^ | 1.47±0.39^b^ | 1.01±0.35^ab^ | 1.43±0.39^b^ | **0.050** | 0.303 | **0.014** |

CS, corn starch diet; WS, wheat starch diet; WF, wheat flour diet

Glucose transporter: *slc5a1*, *solute carrier family 5 member 1*; *slc2a1*, *solute carrier family 2 member 1*; *slc2a2*, *solute carrier family 2 member* 2

Table S7. mRNA levels of selected genes in the liver of three strains of gibel carp fed CS diet, WS diet and WF diet at 6h after the last meal. Data were presented as mean±s.d. (n=3 tanks), statistical differences were evaluated by Two-way *ANOVA* (*p*<0.05, values in bold). In case of interaction, mean values were evaluated by One-way *ANOVA* (*p*<0.05, values in bold) followed by a *post-hoc* Tukey’s test. ^A, B^ Different capital letters indicate significant differences between strains (*p*<0.05). ^a, b^ Different lowercase letters indicate significant differences between diets (*p*<0.05).

| Genes expression | CS | | | WS | | | WF | | | *P*-value | | |
| --- | --- | --- | --- | --- | --- | --- | --- | --- | --- | --- | --- | --- |
|  | Dongting | CASIII | CASV | Dongting | CASIII | CASV | Dongting | CASIII | CASV | Diet | Strain | Diet*Strain |
| mTOR | | | | | | | | | | | | |
| *mtor* | 0.73±0.09 | 0.88±0.23 | 0.83±0.20 | 0.81±0.22 | 0.97±0.15 | 0.74±0.06 | 0.95±0.15 | 1.03±0.34 | 1.22±0.17 | **≤0.001** | 0.101 | 0.119 |
| *eif4ebp1* | 0.78±0.09 | 1.13±0.48 | 1.11±0.27 | 0.97±0.13 | 0.99±0.13 | 0.89±0.13 | 1.04±0.06 | 0.97±0.24 | 1.34±0.32 | 0.098 | 0.062 | 0.052 |
| Glucose transporter | | | | | | | | | | | | |
| *slc2a2* | 0.65±0.16^a^ | 1.37±0.41^b^ | 0.65±0.36^a^ | 0.90±0.09^ab^ | 1.13±0.17^ab^ | 0.98±0.18^ab^ | 0.83±0.15^a^ | 1.10±0.30^ab^ | 1.10±0.24^ab^ | 0.308 | **≤0.001** | **0.019** |
| Glycolysis |  |  |  |  |  |  |  |  |  |  |  |  |
| *gck* | 3.42±1.02 | 6.03±2.66 | 5.08±1.74 | 4.26±1.57 | 4.60±1.80 | 2.88±1.26 | 4.96±2.10 | 5.52±0.41 | 5.15±2.30 | 0.118 | 0.173 | 0.363 |
| *pfkl* | 0.80±0.36^ab^ | 1.17±0.39^abc^ | 1.61±0.23^cd^ | 0.61±0.18^a^ | 1.14±0.24^abc^ | 0.96±0.13^abc^ | 0.78±0.27^ab^ | 1.31±0.54^bcd^ | 1.95±0.30^d^ | **0.002** | **≤0.001** | **0.033** |
| *pklr* | 0.46±0.05^A^ | 1.48±0.23^B^ | 0.68±0.17^A^ | 0.60±0.23^A^ | 1.37±0.33^B^ | 0.80±0.14^A^ | 0.71±0.19^A^ | 1.20±0.40^B^ | 0.87±0.24^A^ | 0.786 | **≤0.001** | 0.160 |
| Gluconeogenesis | | | | | | | | | | | | |
| *pck* | 0.55±0.13^Aa^ | 1.13±0.20^Ba^ | 0.93±0.30^Ba^ | 0.60±0.14^Aa^ | 1.23±0.21^Ba^ | 0.74±0.12^Ba^ | 0.98±0.29^Ab^ | 1.33±0.34^Bb^ | 1.27±0.36^Bb^ | **≤0.001** | **≤0.001** | 0.348 |
| *fbp* | 0.64±0.10 | 1.07±0.22 | 0.95±0.20 | 1.10±0.42 | 0.90±0.12 | 1.09±0.24 | 0.85±0.29 | 0.98±0.29 | 1.03±0.25 | 0.330 | 0.215 | 0.100 |
| *g6p* | 1.06±0.18^AB^ | 1.32±0.17^B^ | 0.69±0.37^A^ | 1.08±0.13^AB^ | 1.23±0.25^B^ | 0.84±0.38^A^ | 0.55±0.16^AB^ | 1.15±0.56^B^ | 0.53±0.24^A^ | 0.103 | **0.005** | 0.746 |
| Lipid synthesis | | | | | | | | | | | | |
| *srebf1* | 0.36±0.20^a^ | 0.72±0.59^a^ | 0.48±0.29^a^ | 0.31±0.14^a^ | 0.82±0.41^a^ | 0.29±0.09^a^ | 2.17±1.41^b^ | 0.97±0.66^b^ | 1.97±1.51^b^ | **≤0.001** | 0.949 | 0.090 |
| *acac* | 0.38±0.28^a^ | 0.73±0.53^a^ | 0.49±0.51^a^ | 0.26±0.18^a^ | 0.75±0.35^a^ | 0.86±0.58^a^ | 1.03±0.69^b^ | 0.90±0.62^b^ | 1.40±0.74^b^ | **0.010** | 0.174 | 0.454 |
| *acly* | 0.61±0.52^a^ | 0.97±0.37^a^ | 0.50±0.09^a^ | 0.38±0.23^a^ | 1.06±0.85^a^ | 0.26±0.06^a^ | 2.02±0.92^b^ | 1.41±0.79^b^ | 1.85±0.90^b^ | **≤0.001** | 0.471 | 0.136 |
| *fasn* | 0.42±0.34^a^ | 0.30±0.11^a^ | 0.24±0.19^a^ | 0.21±0.09^a^ | 0.33±0.11^a^ | 0.39±0.26^a^ | 2.28±1.11^b^ | 0.59±0.64^a^ | 0.72±0.36^ab^ | **0.003** | 0.088 | **0.044** |
| Fatty acid oxidation | | | | | | | | | | | | |
| *cpt1a* | 1.16±0.21 | 1.50±0.15 | 1.42±0.32 | 1.25±0.23 | 1.25±0.26 | 1.03±0.18 | 1.20±0.22 | 1.37±0.43 | 1.48±0.27 | 0.102 | 0.201 | 0.169 |
| *acox3* | 0.80±0.05^A^ | 0.97±0.10^A^ | 1.01±0.49^B^ | 0.99±0.30^A^ | 0.87±0.09^A^ | 1.11±0.14^B^ | 0.93±0.11^A^ | 0.89±0.30^A^ | 1.25±0.24^B^ | 0.486 | **0.018** | 0.480 |

CS, corn starch diet; WS, wheat starch diet; WF, wheat flour diet

mTOR: *mtor*, *mammalian target of rapamycin*; *eif4ebp1*, *eukaryotic translation initiation factor 4E-binding protein 1*

Glucose transporter: *slc2a2*, *solute carrier family 2 member 2*

Glycolysis : *gck*, *glucokinase*; *pfkl*, *6-phosphofructokinase*, *liver type*; *pklr*, *pyruvate kinase*, *liver type*

Gluconeogenesis: *pck*, *phosphoenolpyruvate carboxykinase*; *fbp*, *fructose 1,6-bisphosphatase*; *g6p*, *glucose-6-phosphatase*

Lipid synthesis: *srebf1*, *sterol regulatory element binding transcription factor 1*; *acac*, *acetyl-CoA carboxylase*; *acly*, *ATP citrate lyase*; *fasn*, *fatty acid synthase*

Fatty acid oxidation: *cpt1a*, *carnitine palmitoyl transferase 1 isoform* *alpha*; *acox3*, *acyl-CoA oxidase 3*

Table S8. mRNA levels of selected genes in the muscle of three strains of gibel carp fed CS diet, WS diet and WF diet at 6h after the last meal. Data were presented as mean±s.d. (n=3 tanks), statistical differences were evaluated by Two-way *ANOVA* (*p*<0.05, values in bold). In case of interaction, mean values were evaluated by One-way *ANOVA* (*p*<0.05, values in bold) followed by a *post-hoc* Tukey’s test. ^A, B^ Different capital letters indicate significant differences between strains (*p*<0.05). ^a, b^ Different lowercase letters indicate significant differences between diets (*p*<0.05).

| Genes expression | CS | | | WS | | | WF | | | *P*-value | | |
| --- | --- | --- | --- | --- | --- | --- | --- | --- | --- | --- | --- | --- |
|  | Dongting | CASIII | CASV | Dongting | CASIII | CASV | Dongting | CASIII | CASV | Diet | Strain | Diet*  Strain |
| Glucose transporter | | | | | | | | | | | | |
| *slc2a4* | 2.34±0.72^AB^ | 1.95±0.65^A^ | 3.13±0.84^B^ | 3.00±1.63^AB^ | 1.48±0.58^A^ | 3.55±1.11^B^ | 2.36±0.75^AB^ | 2.42±0.91^A^ | 2.89±0.84^B^ | 0.798 | **0.018** | 0.249 |
| Glycolysis |  |  |  |  |  |  |  |  |  |  |  |  |
| *hk* | 1.69±0.28^B^ | 0.76±0.23^A^ | 1.25±0.30^A^ | 1.71±0.65^B^ | 0.72±0.24^A^ | 0.93±0.42^A^ | 1.46±0.37^B^ | 1.09±0.42^A^ | 1.26±0.35^A^ | 0.466 | **≤0.001** | 0.199 |
| *pfkm* | 1.25±0.36 | 0.94±0.47 | 1.18±0.25 | 1.45±0.56 | 0.96±0.37 | 0.95±0.40 | 1.09±0.28 | 1.00±0.32 | 0.94±0.27 | 0.578 | 0.058 | 0.525 |
| *pkm* | 1.46±0.21^B^ | 1.26±0.28^A^ | 1.51±0.31^AB^ | 1.67±0.69^B^ | 1.19±0.25^A^ | 1.16±0.39^AB^ | 1.59±0.37^B^ | 1.33±0.35^A^ | 1.49±0.16^AB^ | 0.564 | **0.044** | 0.444 |
| Fatty acid oxidation | | | | | | | | | | | | |
| *cpt1a* | 1.35±0.40^B^ | 1.01±0.32^A^ | 1.10±0.29^A^ | 1.52±0.72^B^ | 0.90±0.21^A^ | 0.76±0.27^A^ | 1.23±0.48^B^ | 1.09±0.40^A^ | 0.94±0.30^A^ | 0.762 | **0.005** | 0.437 |
| *acox3* | 1.33±0.32^B^ | 0.87±0.27^A^ | 1.79±0.58^B^ | 1.52±0.72^B^ | 0.79±0.27^A^ | 1.29±0.51^B^ | 1.15±0.25^B^ | 0.93±0.28^A^ | 1.30±0.40^B^ | 0.366 | **≤0.001** | 0.231 |

CS, corn starch diet; WS, wheat starch diet; WF, wheat flour diet

Glucose transporter: *slc2a4*, *solute carrier family 2 member 4*

Glycolysis : *hk*, *hexokinase*; *pfkm*, *6-phosphofructokinase*, *muscle type*; *pkm*, *pyruvate kinase*, *muscle type*

Fatty acid oxidation: *cpt1a*, *carnitine palmitoyl transferase 1 isoform alpha*; *acox3*, acyl-CoA oxidase 3
