# Supplementary material for: Data Mining Evidences Variabilities in Glucose and Lipid Metabolism among Fish Strains: A Case Study on Three Genotypes of Gibel Carp Fed by Different Carbohydrate Sources
Source: Aquac Nutr. 2023 Feb 2;2023:7589827. doi: 10.1155/2023/7589827 (PMC9973145; doi:10.1155/2023/7589827)
Supplement: Supplementary Materials — To compare the differences in detail and make the comparison clear, we also performed statistical analysis on all the results, which was shown in the Supplementary Data. Accordingly, the statistical results were described and showed in the Supplementary Result. [file 7589827.f1.zip › Supplementary Result (1).docx]

**3.3 Supplementary Result**

S3.3.1 The difference of growth performance by statistical analysis

After 8 weeks, growth performance of three strains of gibel carp fed with different carbohydrate sources were obtained. Regarding the effect of diets, feed efficiency (FE), protein retention efficiency (PRE) and lipid retention efficiency (LRE) were significantly higher in trout fed WF diet (**Table S3**, *p*<0.05), and they were also significant different between strains: F strain had higher FE than A strain followed by DT strain (*p*<0.05); PRE and LRE was higher in F strain than DT strain (*p*<0.05), but not significant different with A strain (*p*>0.05). There was a significant interaction of strains and diets for specific growth rate (SGR) (*p*<0.05), F strain fed with WF diet had higher SGR than other groups (*p*<0.05). Hepatosomatic index (HSI) and condition factor (CF) were significant different between strains. A strain had higher HSI than F strain, then followed by DT strain (*p*<0.05), while there was no difference of HSI when fed with different diets (*p*>0.05). F strain and DT strain had higher CF than A strain (*p*<0.05), while no difference of CF was observed when the fish fed with different diets (*p*>0.05).

S3.3.2 The difference of whole-body and tissue composition, intestinal amylase by statistical analysis

Whole body and tissue composition, amylase were shown in**Table S4**. Whole body protein, lipid, liver lipid and muscle glycogen were significantly affected by strains (*p*<0.05). A strain had higher body protein than DT strain and F strain (*p*<0.05). F strain showed lower body and liver lipid content (*p*<0.05), but had higher muscle glycogen compared with DT strain and A strain (*p*<0.05). Liver lipid was also higher in fish fed with WF diet (*p*<0.05). There was a diet*strain interaction for liver glycogen that A strain showed higher liver lipid when fed with CS diet and WS diet, but it was lower in WF diet (*p*<0.05). Amylase activity was affected by both fish strain and diets. A strain and F strain had higher amylase activity than DT strain (*p*<0.05), and amylase activity was higher in the fish when fed with WS diet and WF diet than fed with CS diet (*p*<0.05).

S3.3.3 The difference of post-prandial plasma metabolites levels by statistical analysis

Plasma glucose, triglyceride and cholesterols were measured at 6h after the last meal (**Table S5**). Significant interactions of diets and strains for plasma glucose and triglycerides were observed (*p*<0.05), F strain fed with WF diet showed lower plasma glucose but higher triglyceride (*p*<0.05). Total cholesterol, LDL cholesterol and HDL cholesterol were higher in F strain and A strain than DT strain (*p*<0.05), and higher in WS diet followed by CS diet than WF diet (*p*<0.05).

3.3.4 The difference of metabolism in the intestine by statistical analysis

The transcriptional results of *slc5a1, slc2a1* and *slc2a2* are shown in **Table S6***.* Gene expression of *slc5a1* and *slc2a1* was significantly affected by fish genotype*. slc5a1* was more highly expressed in CASⅤ than in CASⅢ (*p*<0.05). Dongting showed higher expression of *slc2a1* than CASⅢ and CASⅤ (*p*<0.05). There was an interaction between CASⅤ and carbohydrate source for the expression of *slc2a2* (*p*<0.05). CASⅤ fed the WF diet showed significantly higher expression of *slc2a2*, but when fed the WS diet, CASⅤ showed significantly lower expression of *slc2a2*.

3.3.5 The difference of metabolism in liver by statistical analysis

There was a diet*strain interaction for the gene expression of glucose transporter 2 (**Table S7**, *p*<0.05). CASⅢ showed higher expression of *slc2a2* than Dongting and CASⅤ when fed the CS diet (*p*<0.05), whereas there was no significant difference in *slc2a2* expression between the three strains when fish were fed the WS and WF diets (*p*>0.05). Regarding glucose metabolism in the liver, the mRNA levels of target genes encoding key enzymes involved in glycolysis were analysed (**Table S7**). A diet*strain interaction was found at the mRNA level for *pfkl* (*p*<0.05): Dongting fed the WS diet showed significantly lower expression of *pfkl,* whereas *pfkl* was more highly expressed in CASⅤ fed the WF diet (*p*<0.05). CASⅢ had a higher mRNA level of *pklr* than Dongting and CASⅤ (*p*<0.05). The mRNA levels of key gluconeogenic enzymes were studied (**Table S7**). When fed the WF diet, gibel carp exhibited a significant increase in the expression of *pck* (*p*<0.05), and *pck* was more highly expressed in CASⅢ and CASⅤ than in Dongting (*p*<0.05). CASⅢ showed higher expression of *g6p*, followed by CASⅢ and then CASⅤ (*p*<0.05). We also analysed lipid metabolism in the liver, including lipid synthesis and fatty acid oxidation (**Table S7**). The WF diet was associated with increased mRNA levels of *srebf1*, *acac* and *acly* (*p*<0.05), and a diet*strain interaction was observed for *fasn*: Dongting fed the WF diet had a higher transcriptional level of *fasn* than the other groups (*p*<0.05). CASⅤ showed higher expression of *acox3*, a fatty acid oxidation gene, than Dongting and CASⅢ, irrespective of the diet.

3.3.6 The difference of metabolism in muscle by statistical analysis

As the main glucose transporter in fish muscle, *slc2a4* was differentially expressed between strains (**Table S8**, *p*<0.05), and CASⅤ had higher *slc2a4* expression than CASⅢ (*p*<0.05). For glycolysis in the muscle, *hk* was more highly expressed in Dongting, and *pkm* was more highly expressed in Dongting, followed by CASⅤ and CASⅢ (*p*<0.05). The mRNA levels of *cpt1a* and *acox3*, which are involved in fatty acid oxidation, were significantly different between strains (*p*<0.05). The gene expression of *cpt1a* was higher in Dongting than in CASⅢ and CASⅤ (*p*<0.05). Dongting and CASⅤ had higher expression of *acox3* than CASⅢ (*p*<0.05).
